# Transcutaneous Auricular Neurostimulation Modulates Pain Perception in Survivors of Stroke With Chronic Upper-Extremity Pain: A Randomized, Sham-Controlled Pilot Study

**DOI:** 10.1016/j.neurom.2025.12.005

**Published:** 2026-01-16

**Authors:** Xiaolong Peng, Stewart S. Cox, Brenna Baker-Vogel, Fisayo Omonije, Katherine Tucker, Bailey Huttig, Falon Sutton, Nicole Cash, Marion Wood, Steven A. Kautz, Bashar W. Badran, Jeffrey J. Borckardt

**Affiliations:** 1 Department of Psychiatry and Behavioral Sciences, Medical University of South Carolina, Charleston, SC, USA; 2 Ralph H. Johnson VA Medical Center, Charleston, SC, USA; 3 Department of Health Sciences and Research, College of Health Professions, Medical University of South Carolina, Charleston, SC, USA; 4 Division of Physical Therapy, Department of Rehabilitation Sciences, College of Health Professions, Medical University of South Carolina, Charleston, SC, USA

**Keywords:** Chronic pain, neuromodulation, QST, stroke, Tan

## Abstract

**Background:**

Chronic pain is a common and debilitating consequence of stroke. Although pharmacologic interventions are typically the first-line treatment, they often have limited efficacy and carry risks such as side effects and dependence. Evidence for neuromodulation therapies is positive but limited owing to high cost and invasiveness. Therefore, developing noninvasive, affordable neuromodulation approaches for treating poststroke pain could offer significant clinical benefits. Transcutaneous auricular neurostimulation (tAN), an affordable and wearable neuromodulation technique, has shown promise for analgesia, but research specific to poststroke pain remains limited.

**Objectives:**

This pilot study aims to explore the safety/feasibility, in addition to the analgesic effects, of tAN in patients with stroke with chronic upper-extremity pain.

**Materials and Methods:**

Overall, 15 patients with stroke with chronic upper-extremity pain were enrolled. Each participant received a single 30-minute session of tAN (active or sham). Subjective stroke-related pain ratings and thermal pain thresholds (through quantitative sensory testing; QST) were collected immediately before and after the 30-minute tAN session. Repeated-measures two-way analyses of variance were performed with group (sham vs active tAN) and time (pre- vs post-tAN) as factors to evaluate the potential analgesic effects of tAN on both pain measures.

**Results:**

QST pain threshold analysis in the 14 patients who completed the study revealed a significant time × group interaction [F(1,12) = 7.658, *p* = 0.017]. Post hoc analysis indicated a potentiation in pain threshold after active stimulation (*p* = 0.032), whereas no change was observed in the sham group.

**Conclusions:**

This pilot study showed that tAN is safe and feasible for patients with chronic poststroke pain. Although subjective pain scores improved in both groups, pain threshold increased significantly in the active group compared with the sham. Future studies should aim to assess long-term efficacy and optimized parameters, dosing, and treatment duration of tAN for this clinical population to aid in poststroke pain.

**Clinical Trial Registration:**

The Clinicaltrials.gov registration number for the study is NCT06456385.

## INTRODUCTION

In the United States, there are nearly 800,000 individuals annually who experience a stroke, making it a leading cause of long-term disability.^1^ Up to 70% of survivors of stroke experience poststroke pain, such as spasticity, complex regional pain syndrome, and central poststroke pain.^2,3^ The first line of treatment for poststroke pain (PSP) involves pharmacologic approaches; however, many commonly used medications, including antidepressants such as amitriptyline, anti-inflammatory medications, and antiepileptic drugs such as gabapentin and pregabalin, have shown mixed efficacy.^2-4^ Moreover, patients whose condition is resistant to initial treatments often require opioids for pain management, which carries a risk of dependency,^5,6^ nor have they consistently been shown to be beneficial in the management of chronic PSP.^7^

Emerging evidence suggests that neuromodulation techniques, such as deep brain stimulation (DBS) and vagal nerve stimulation (VNS), could be beneficial for pain refractory to traditional treatments. DBS has been studied across numerous neuropathologies for its potential analgesic properties. For example, DBS has been shown to reduce subjective pain symptoms in patients with spinal cord injury,^8,9^ Parkinson’s disease,^10,11^ and chronic neuropathic pain.^12-15^ Although there is large heterogeneity regarding the efficacy across these different neuropathologies, there appears to be promise in the procedure’s ability to improve patients’ affective component of pain.^16^ Similarly, VNS, a procedure initially used for treatment-resistant epilepsy, also has indicated robust analgesic effects in previous studies involving chronic pain disorders including fibromyalgia, pelvic pain syndrome, and headaches.^17-20^ Proposed mechanisms include inhibition of spinal nociceptive pathways, and anti-inflammatory properties. However, despite their promise, these interventions are often impractical for wide-spread clinical use owing to their high cost and invasive nature. As such, there is a critical need for accessible, noninvasive, and affordable alternatives.

One such option that has gained increasing notoriety is transcutaneous auricular neurostimulation (tAN). This noninvasive neurostimulation approach targets the auricular branch of the vagus nerve through electrodes placed on the cymba conchae and the trigeminal nerve of the outer ear.^21-23^ tAN has indicated therapeutic potential in several neuropsychiatric conditions, including epilepsy, depression, poststroke motor recovery, and spasticity reduction.^24-29^ Moreover, previous studies indicate that tAN can modulate conditioned pain responses in healthy subjects after a single session of stimulation^30^ and may have analgesic effects in conditions such as migraine.^17,21,24^ However, to our knowledge, no studies to date have explored the feasibility and analgesic efficacy of tAN in PSP.

In this randomized, sham-controlled, single-blind pilot trial, we investigated the safety and feasibility of implementing tAN in patients with stroke experiencing upper-extremity (UE) PSP. We also assessed changes in pain and sensory experience to a thermal stimulus through subjective pain scales and quantitative sensory threshold (QST) testing, which systematically determines information about Aβ fibers (sensory), Aδ fibers (pain), and C fibers (pain tolerance).^31,32^ These pain assessments were performed before and after a single 30-minute tAN stimulation session, and active and sham groups were compared.

## MATERIALS AND METHODS

### Study Overview

In this study, participants with chronic UE PSP were recruited through the Registry for Stroke Recovery at the Medical University of South Carolina (MUSC) to evaluate the effects of tAN on chronic PSP. Each participant underwent a single experimental visit, during which a single 30-minute tAN session was administered. Pain measures were assessed before and after the tAN treatment. Written informed consent was obtained from all participants. This study was approved by the MUSC institutional review board and is registered on ClinicalTrials.gov (NCT06456385).

### Participants

A total of 15 patients with stroke (mean age: 56.73 years; ten women) with chronic UE PSP were enrolled in this study. The overall demographic and clinical characteristics of all participants are listed in Table 1. The inclusion criteria were 1) aged between 18 and 80 years, 2) a history of ischemic or hemorrhagic stroke ≥six months before recruitment, and 3) unilateral lesions in the left hemisphere with right UE pain. Exclusion criteria included 1) primary intracerebral hematoma or subarachnoid hemorrhage, 2) a documented history of dementia, 3) a documented history of uncontrolled depression or other psychiatric comorbidity, 4) uncontrolled hypertension despite treatment, defined as systolic blood pressure (SBP)/diastolic blood pressure (DBP) ≥180/100 mmHg at baseline, and 5) pregnancy.

### tAN Procedure

**Figure 1.**
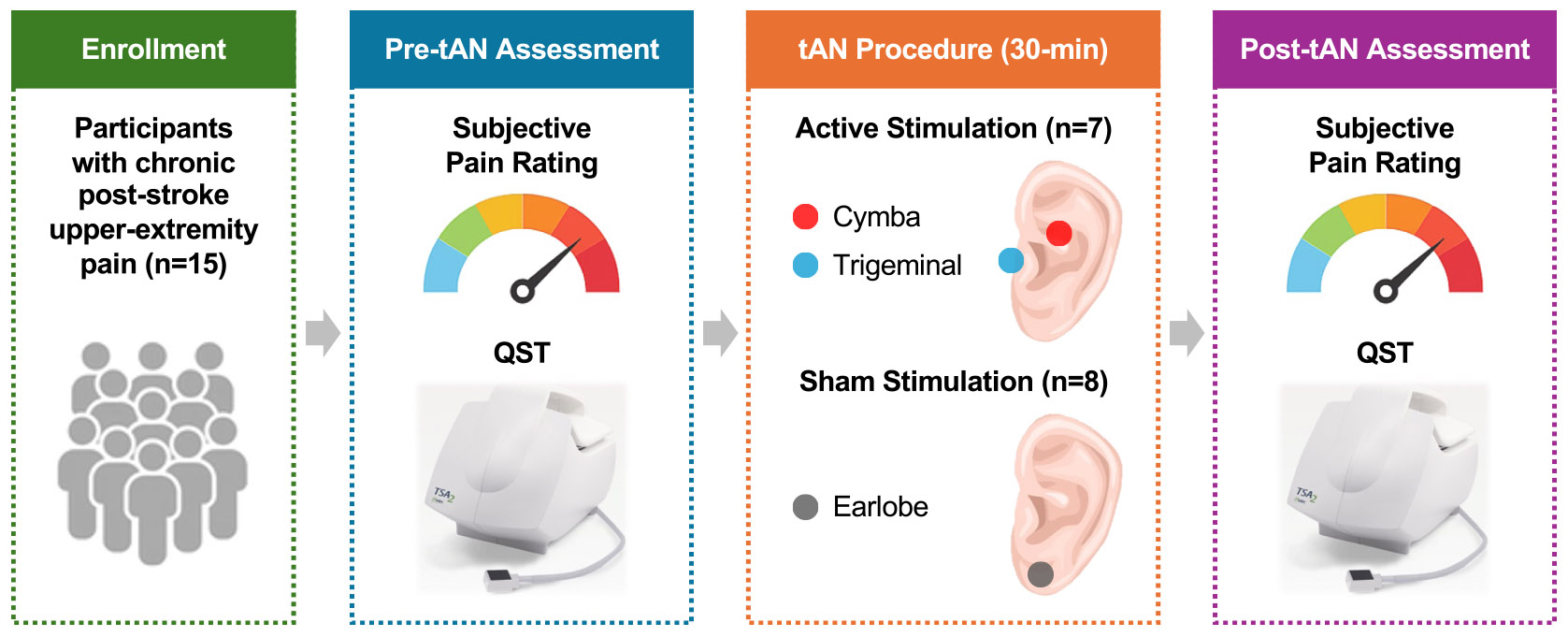
Overview of the experimental workflow. A total of 15 participants with chronic UE PSP were enrolled in this randomized, sham-controlled pilot study. At baseline, participants completed a pre-tAN pain assessment, which included both QST testing and subjective pain ratings. They were then randomly assigned to receive a single 30-minute tAN session of either active stimulation targeting the cymba conchae and trigeminal nerve, or sham stimulation applied to the earlobe. After the stimulation session, participants underwent post-tAN QST testing and subjective pain ratings.

On enrollment, participants were assigned to the intervention groups (Active vs Sham) using a computer-generated allocation sequence. Participants were instructed only that they would receive ear stimulation and were blinded to their group assignment. They received either the “Active” tAN stimulation at the cymba concha and trigeminal nerve (Fig. 1; *n* = 7) or “Sham” stimulation on the earlobe (*n* = 8). All stimulation was administered using the Sparrow Ascent Transcutaneous Auricular Neurostimulation device (Spark Biomedical; Dallas, TX).

Participants first underwent a perceptual threshold (PT) assessment to determine appropriate stimulation intensity using a standardized procedure previously described.^28,29,33,34^ Briefly, the skin on and around the outer ear was cleaned with a sterile alcohol preparation pad to ensure maximal electrode adhesion. Electrodes were then placed on either the earlobe, the anterior to the tragus (tongue-like projection of cartilage anterior to the ear canal), or the cymba concha (the upper, smaller area of the hollow conchal bowl above the crus of the helix; Supplementary Data Fig. S1 presents actual electrode placement). After attaching the electrode to the ear, the current intensity (mA) was gradually increased until the participant first perceived the stimulation. This minimum detectable electrical current intensity was recorded as the PT. The stimulation intensity was then set to 2 × PT for the tAN procedure. If a participant reported the stimulation intensity as uncomfortable, it would be gradually reduced until a comfortable level was achieved. The tAN session lasted for 30 minutes. For active stimulation, two stimulation frequencies were applied: 15 Hz at the cymba concha and 100 Hz anterior to the trigeminal nerve, with a pulse duration of 250 μs. For sham stimulation, identical stimulation parameters to those for cymba concha were applied; however, the electrodes were placed on the earlobe, an area with low cranial nerve innervation and minimal expected neuromodulatory effects.^35^

### Pain Assessment

In this study, we collected two types of pain measures: subjective ratings of stroke-related pain and thermal pain thresholds assessments (QST), administered immediately before and after the tAN procedure. For subjective pain rating, participants self-reported their current UE PSP using a standard numeric pain rating scale (0–10), when 0 equals “no pain” and 10 equals “worst pain imaginable.” For QST, a cutaneous thermal stimulus was delivered to the left volar forearm (the side unaffected by stroke) of participants, approximately 5 cm from the wrist, using a 30 × 30 mm advanced thermal stimulator thermode from the Medoc Pathway System (Medoc Advanced Medical Systems Ltd, Durham, NC). The unaffected arm was selected for QST to ensure that the pain stimulus could be safely tolerated and any tAN-related changes in thermal pain thresholds could be accurately recorded while minimizing the influence of stroke-related sensory deficits on assessment outcomes. The thermal stimulus was gradually increased from 32 °C at a rate of 0.5 °C per second. Participants were instructed to press a button when the stimulus was first detected (sensory threshold), painful (pain threshold), and intolerable (pain tolerance threshold). This procedure was repeated five times per QST session with a 20-second intertrial interval. The pain assessment data were analyzed by individuals who were independent from the research coordinators who conducted the experiment.

### Statistical Analysis

Demographic statistics were compared between active and sham tAN using unpaired two-sample *t*-tests. For the QST data, th first of each of the five trials was discarded to avoid novelty and orienting effects, and to ensure consistent Aδ fiber activation suppression during each testing period.^31,32^ Data from the remaining four trials of each condition were averaged to represent the final sensory, pain, and tolerance thresholds. Repeated-measures two-way analyses of variance (ANOVAs) were conducted to evaluate changes in average QST sensory, pain, and threshold temperatures over time, with group (sham vs active tAN and time (pre- vs post-tAN) as factors. The same analysis was applied to the subjective pain scale. Post hoc analyses were performed using Holm-Sidak’s correction for family-wise error, with α set at 0.05. In addition, to assess individual changes over time, the percentage change ($\frac{post-pre}{pre}\times 100$) was calculated for each participant’s average QST sensory, pain, and threshold temperatures, in addition to subjective pain ratings. Unpaired two-sample *t*-tests were then used to compare these changes between the sham and active groups. All analyses were conducted using Prism Software version 10 (GraphPad Software; Boston, MA), and data are expressed as mean ± SD.

## RESULTS

### Pain Location in Participants With Chronic PSP

**Figure 2.**
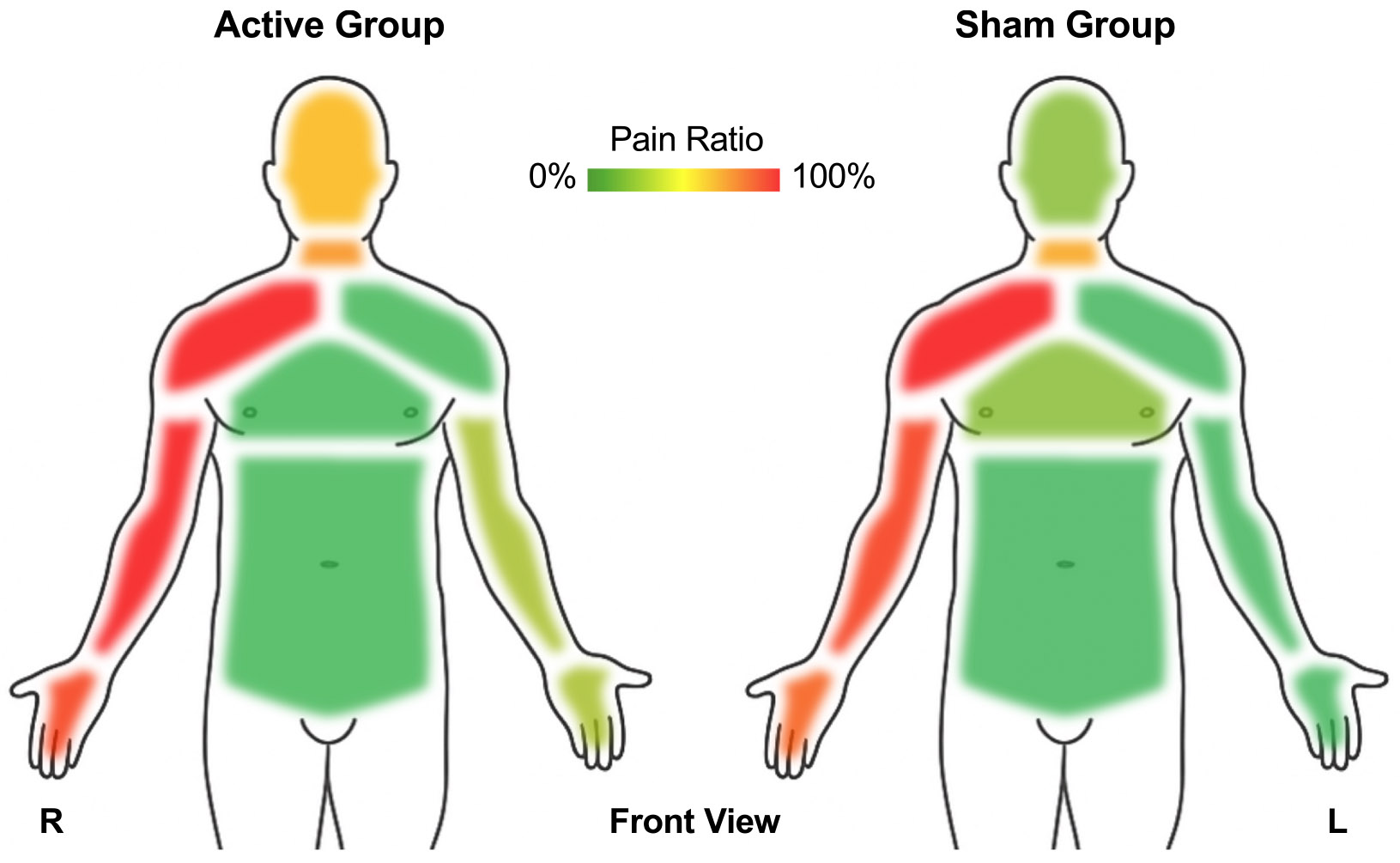
Spatial distribution of chronic PSP. At baseline, participants completed a pain questionnaire indicating whether they experienced pain in upper body regions, including the head, neck, chest, abdomen, and UE: left and right shoulders, arms, and hands. For each region, the proportion of participants reporting pain was calculated within each group. The spatial distribution of chronic PSP was consistent between the two groups, and all participants reported right-sided UE pain contralateral to their stroke.

All participants completed a baseline questionnaire to indicate the locations of their chronic PSP in the upper body, including the head, neck, chest, abdomen, and the left and right UEs (shoulders, arms, and hands). The proportion of participants reporting pain in each region was calculated within each group. The spatial patterns of pain distribution were consistent between the two groups (Fig. 2; Spearman correlation, *r* = 0.863, *p* = 0.001), and all participants reported experiencing pain in the right UE (active group: right shoulder = 100%, right arm = 100%, right hand = 86%; sham group: right shoulder = 100%, right arm = 88%, right hand = 75%). Data from one participant in the sham group were excluded from analysis owing to a misunderstanding of the QST procedure, which yielded a reported higher pain threshold than tolerance threshold.

### PT and Stimulation Intensity of tAN

PT varied between tAN target sites (cymba conchae: 1.66 ± 0.77 mA; trigeminal nerve: 1.84 ± 0.84 mA; earlobe: 1.16 ± 0.45 mA). All participants received ear stimulation at twice their PT unless they stated it was uncomfortable, in which case a reduced stimulation intensity was applied. The final mean stimulation intensities were as follows: cymba conchae: 3.22 ± 1.46 mA; trigeminal nerve: 3.14 ± 1.01 mA; earlobe: 2.27 ± 0.84 mA. One-way ANOVAs were performed and indicated that there were no significant differences in PT [F (2,19) = 1.74, *p* = 0.203] or in stimulation intensity [F (2,19) = 1.52, *p* = 0.250] across stimulation sites.

### Quantitative Sensory Testing

**Figure 3.**
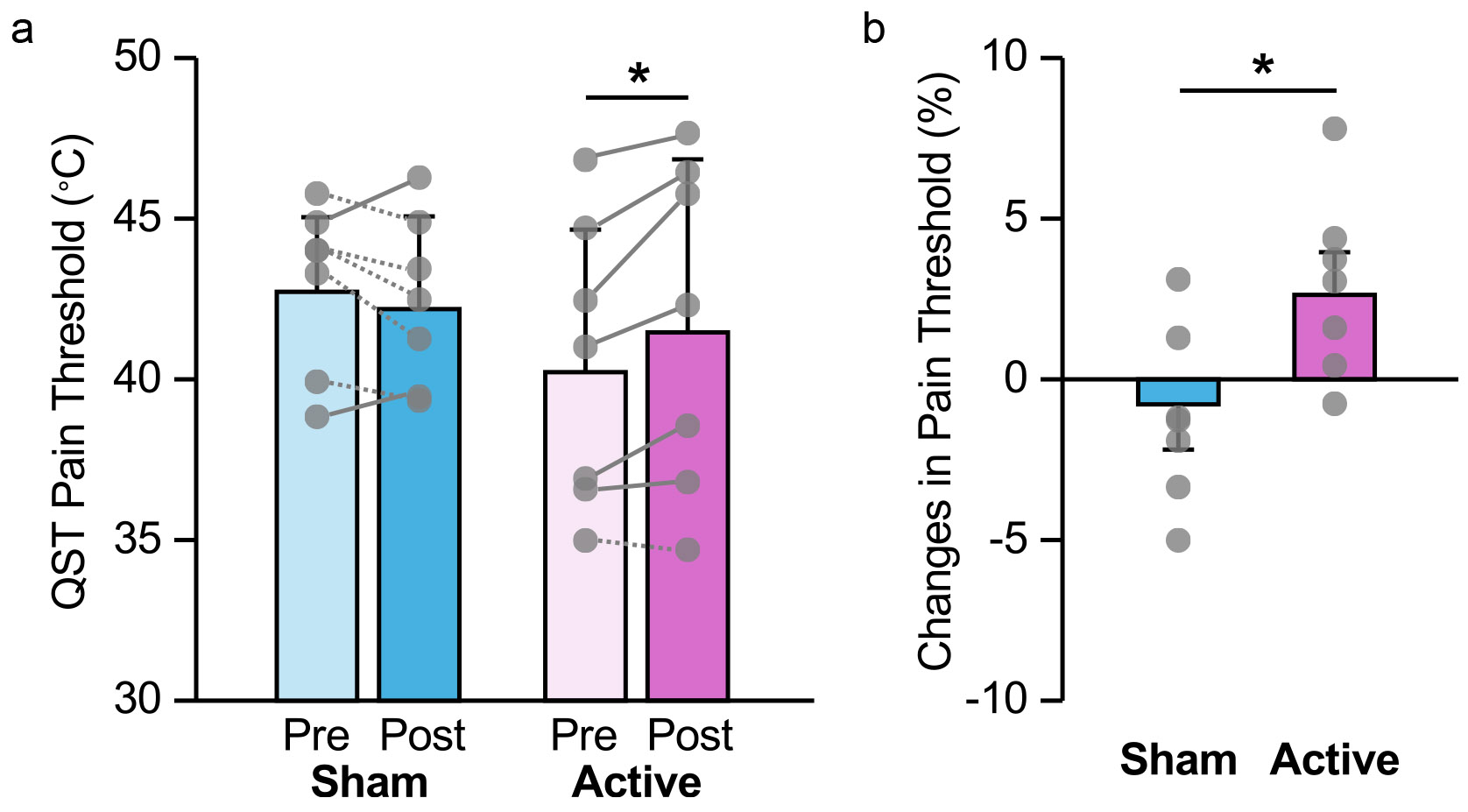
A single session of active tAN improves thermal pain threshold in individuals with chronic PSP. a. A repeated-measures two-way ANOVA on thermal pain threshold revealed no significant main effect of time [F (1,12) = 1.407, *p* = 0.259] or group [F (1,12) = 0.571, *p* = 0.464], but a significant group × time interaction was observed [F (1,12) = 7.658, *p* = 0.017]. Post hoc paired *t*-test analyses indicated a significant increase in thermal pain threshold after active tAN compared with baseline [*t* (12) = 2.795, *p* = 0.032], whereas no significant change was observed in the sham group [*t* (12) = 1.118, *p* = 0.285]. b. Analysis of the percentage change ($\frac{post-pre}{pre}\times 100$) in thermal pain threshold revealed a significantly greater increase in the active group than in the sham group [two-sample *t*-test, *t* (12) = 2.777, *p* = 0.017]. Solid and dashed lines indicate increased or decreased QST pain thresholds, respectively. * *p* < 0.05.

To assess whether tAN modulates pain perception, we analyzed thermal pain thresholds (the temperature at which participants reported the stimulus as painful) before and after stimulation. Before analysis, normality was assessed using the D’Agostino-Pearson test. The data set met normality assumptions (*p* = 0.23). Next, a repeated-measures two-way ANOVA was conducted and revealed no significant main effects of time [F (1,12) = 1.407, *p* = 0.259] or group [F (1,12) = 0.571, *p* = 0.464]. However, a significant group × time interaction was observed [Fig. 3a; F (1,12) = 7.658, *p* = 0.017], indicating differential changes between the active and sham groups. Post hoc paired *t*-tests showed that participants in the active group had a significant increase in thermal pain threshold after tAN compared with baseline [*t* (12) = 2.795, *p* = 0.032], whereas no significant change was observed in the sham group [*t* (12) = 1.118, *p* = 0.285]. A two-sample *t*-test was performed and revealed a significantly greater percentage increase in thermal pain threshold in the active group than in the sham group [Fig. 3b; two-sample *t*-test, *t* (12) = 2.777, *p* = 0.017].

Moreover, thermal sensory threshold (the temperature at which participants first felt the thermal stimulus) and pain tolerance (the temperature at which participants could no longer tolerate the stimulus) were assessed before and after stimulation in both the active and sham groups. For sensory threshold, a repeated-measures two-way ANOVA revealed a significant main effect of time [Supplementary Data Fig. S2a; F (1,12) = 4.782, *p* = 0.042], but no significant main effect of group [F (1,12) = 2.713, *p* = 0.126] or a group × time interaction [F (1,12) = 0.042, *p* = 0.841]. A comparison of percentage change in sensory threshold showed no significant difference between the active and sham groups [Supplementary Data Fig. S2b; *t* (12) = 0.346, *p* = 0.734]. For pain tolerance, no significant effects of group [Supplementary Data Fig. S2c; F (1,12) = 0.682, *p* = 0.425], time [F (1,12) < 0.001, *p* = 0.989], or group × time interaction [F (1,12) = 0.048, *p* = 0.831] were observed. Similarly, there was no significant difference in percentage change in pain tolerance between groups after stimulation [Supplementary Data Fig. S2d; *t* (12) = 0.234, *p* = 0.819].

### Subjective Stroke-Related Pain Rating

Subjective pain ratings were assessed before and after stimulation. Data normality was assessed and found to pass the D’Agostino-Pearson test (*p* = 0.11). The repeated-measures ANOVA showed a significant main effect of time [Supplementary Data Fig. S3a; F (1,12) = 10.110, *p* = 0.008], indicating that both groups reported reduced stroke-related pain after the session. However, there was no significant main effect of group [F (1,12) = 0.114, *p* = 0.742] or a group × time interaction [F (1,12) < 0.001, *p* > 0.999]. A two-sample *t*-test comparing the percentage change in subjective pain ratings between the active and sham groups also found no significant difference between groups [Supplementary Data Fig. S3b; *t* (12) = 0.185, *p* = 0.857].

### Safety and Tolerability

In this study, tAN stimulation was well tolerated by all participants (*N* = 15), with no adverse events reported. Moreover, all participants completed the full 30-minute tAN session without any changes in blood pressure [SBP mean ± SD, pre-tAN: 123.6 ± 13.9 mmHg, post-tAN: 123.5 ± 17.7, paired *t*-test, *t* (14) = 0.024, *p* = 0.981; DBP mean ± SD, pre-tAN: 83.7 ± 9.0 mmHg, post-tAN: 84.7 ± 9.1 mmHg, *t* (14) = 0.596, *p* = 0.561]. These findings suggest that tAN is a safe and well-tolerated intervention for individuals with chronic PSP.

## DISCUSSION

In this study, we performed a sham-controlled pilot study to investigate whether tAN can modulate pain perception in patients with stroke with chronic UE pain. Our findings suggest that tAN is both safe and feasible in this population. Furthermore, even a single 30-minute stimulation session significantly enhanced the antinociceptive effect of thermal pain in the active group compared with sham. Although promising, caution should be exercised owing to the small sample size. To our knowledge, this is the first study to show antinociceptive effects of tAN in individuals with chronic PSP.

Although emerging evidence supports tAN can modulate pain, its efficacy appears to vary depending on the clinical context. Studies in healthy volunteers have yielded mixed results: some report increased pain thresholds^36^ whereas others find no significant difference from sham or placebo.^37,38^ In contrast, findings from pathologic pain populations have been more consistent. For example, pilot trials in individuals with chronic low back pain and osteoarthritis indicated persistent pain relief after tAN.^39,40^ Similar effects were observed in patients with systemic lupus erythematosus, when pain relief was correlated with cumulative stimulation dose.^41^ In addition, tAN has been found to reduce pain and analgesic requirements in perioperative and postoperative settings.^42,43^ Our findings extend this body of research to individuals with PSP, a population that could benefit greatly from tAN. Current pharmacologic treatments for PSP yield a mean pain reduction of approximately 58% according to meta-analytic evidence, leaving a large proportion of patients with residual pain and ongoing decreased psychologic issues and lower quality of life.^7^ Moreover, many individuals with stroke experience chronic motor impairments that limit their mobility and ability to travel for treatment. The tAN device, being portable, easy to set up, noninvasive, and affordable, may therefore be particularly advantageous for patients with stroke living in rural or underserved areas by enabling at-home management of PSP. Our early evidence indicates the safety and feasibility of tAN in individuals with PSP. Future larger-scale studies are needed to evaluate remotely supervised, at-home tAN paradigms for managing PSP. Beyond pain relief, tAN also has shown therapeutic potential for improving motor function and enhancing neuroplasticity after stroke.^28,44,45^ These broader benefits further highlight its potential clinical value for the stroke population.

**Figure 4.**
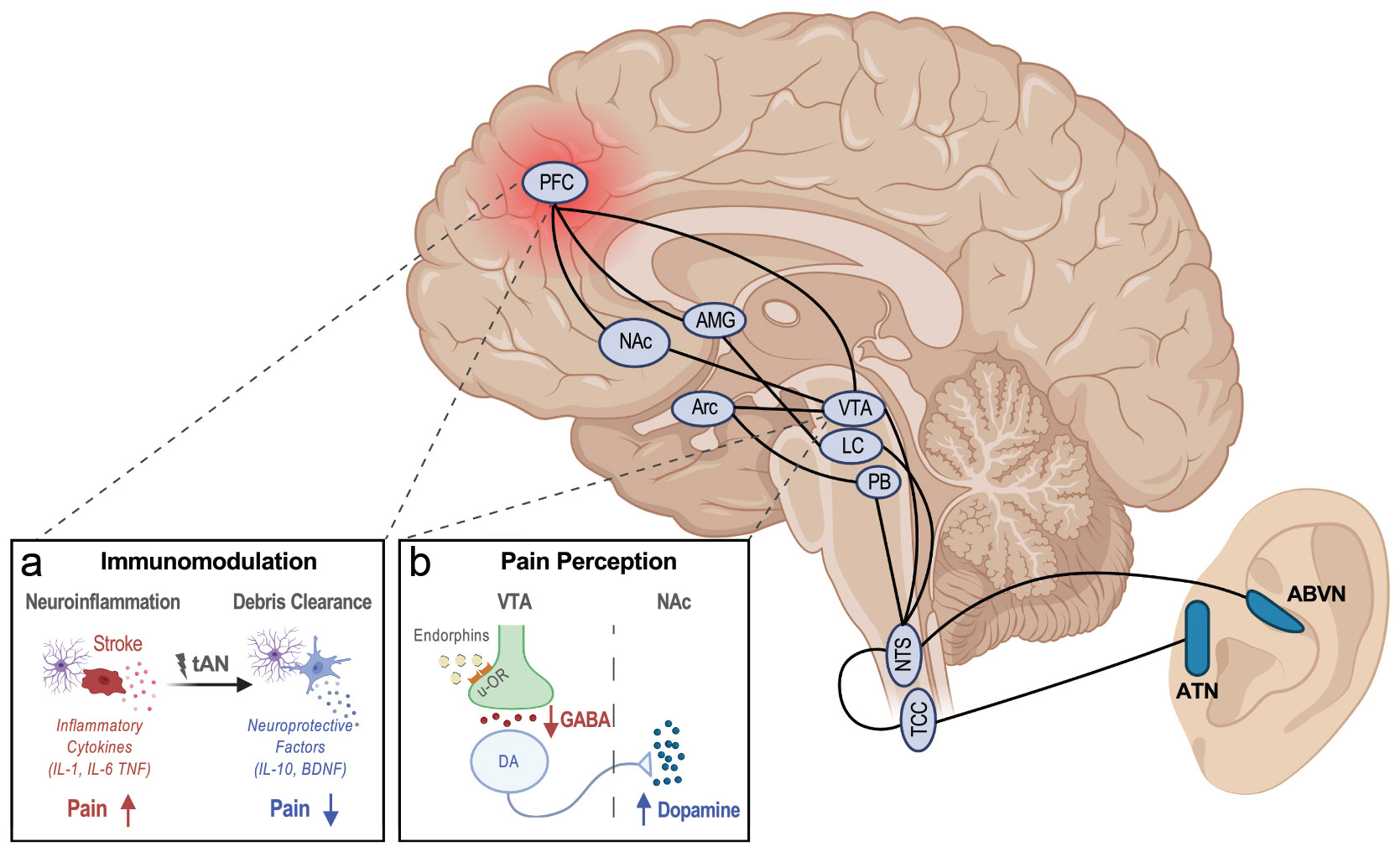
Proposed mechanisms of pain modulation through tAN. This schematic illustrates a current working model of how tAN modulates central pain pathways. Specifically, tAN delivers stimulation to the ABVN and ATN, with signals relayed from brainstem nuclei to higher-order regions involved in pain perception. Stimulation also has been shown to induce endorphin release, which may influence pain signaling through the VTA. More specifically related to the pathology of PSP, tAN has been associated with reductions in proinflammatory cytokines and increases in neuroprotective factors, including IL-4, IL-10, and BDNFs, which have been associated with pain relief. ABVN, auricular branch of the vagus nerve; AMG, amygdala; Arc, arcuate nucleus; ATN, auricular branch of the trigeminal nerve; BDNF, brain-derived neurotrophic factor; DA, dopaminergic neuron; LC, locus coeruleus; NAc, nucleus accumbens; PB, parabrachial nucleus; PFC, prefrontal cortex; TCC, trigeminocervical complex; TNF, tumor necrosis factor; u-OR, mu opioid receptor.

The underlying mechanisms by which tAN reduces PSP in patients remain understudied; however, two potential pathways may contribute to its efficacy (Fig. 4). From a central neuroanatomical perspective, tAN has been shown to activate the nucleus tractus solitarius (NTS), a key brainstem region involved in pain modulation and emotional processing.^46,47^ Activation of the NTS can further influence downstream limbic and pain-related structures, including the amygdala, anterior cingulate cortex, and insula.^48^ One particularly relevant downstream target is the ventral tegmental area (VTA), which is involved in both reward and pain modulation.^49,50^ Activation at the VTA leads to the release of endorphins that inhibit pain transmission, a mechanism supported by findings that opioid antagonists such as naloxone can block the analgesic effects of VNS.^51-53^ Endorphin release in the VTA is hypothesized to modulate pain through disinhibition of dopamine neurons, ultimately increasing dopamine signaling in regions such as the nucleus accumbens,^51,54,55^ which can alter pain perception. A second, more pathology-specific pathway involves the vagus nerve’s anti-inflammatory action through the cholinergic anti-inflammatory reflex, mediated by nicotinic acetylcholine receptors and shown to reduce proinflammatory cytokines such as tumor necrosis factor-α, interleukin (IL)-1β, and IL-6.^56-58^ Both preclinical^59^ and clinical^60,61^ research also suggest that stimulation can increase anti-inflammatory markers such as IL-4 and IL-10, and may support neurogenesis through increases in brain-derived neurotrophic factor.^44^ These stimulation-induced changes have been associated with improved pain outcomes, although the exact mechanisms remain to be fully explored.

This pilot study evaluated QST on the unaffected limb in patients after stroke to ensure safety and to maintain measurement accuracy by avoiding sensory deficits that could impair the evaluation. Given this methodologic choice, the observed increase in pain threshold may reflect a central antinociceptive effect rather than directly modifying pathologic pain inflammatory markers related to stroke, particularly considering the acuity and laterality of pain assessment. Future studies should evaluate QST on both the affected and unaffected limbs to compare the differential impact of tAN. In addition, future studies should investigate whether tAN influences peripheral or central cytokine levels in patients after stroke, and advanced brain imaging techniques could help elucidate ways pain-related networks are modulated by tAN in this population.

Chronic UE PSP can be due to a variety of causes, including shoulder subluxation, muscle weakness, spasticity, or central PSP, a neuropathy that often occurs secondary to damage in the thalamus or other cortical substrates involved in pain perception, such as the primary motor or somatosensory cortices, and posterior insula.^62,63^ In this study, we did not specify the underlying cause of the patient’s UE pain or limit the lesion to a specific brain region. Previous research suggests that there are no significant differences in pain severity or in QST scoring between thalamic and extrathalamic strokes.^64^ Furthermore, preliminary results suggest tAN is helpful in treating both peripheral and central pain conditions;^21,65^ differences in functional connectivity patterns may influence individual responses to stimulation. Further research is warranted to explore these distinctions and their implications for tAN efficacy.

Interestingly, although both groups reported subjective pain improvement, there was no significant difference between the active and sham groups. This may reflect a strong placebo effect, which has been documented in neuromodulatory interventions such as transcranial magnetic stimulation (TMS) in poststroke populations.^66^ Future trials may benefit from including a no-treatment control arm and should be powered to detect clinically meaningful differences. In addition, the single 30-minute stimulation session may have been insufficient to produce sustained clinical effects. There is currently limited research guiding optimal dosing parameters for tAN in PSP,^24,67^ which should be explored in future studies.

### Limitations and Future Directions

This study was an early proof-of-concept pilot trial designed to establish safety and feasibility for tAN in individuals with chronic PSP, and to evaluate its short-term effects on pain management. As such, several limitations should be considered in future studies. First, although we observed a significant increase in nociceptive threshold after a single tAN stimulation, this finding must be interpreted with caution, given the small sample size. Future studies should be adequately powered to more clearly determine the clinical efficacy of tAN for PSP. Moreover, our study included individuals with both hemorrhagic and ischemic strokes. Although both populations have substantial risks of PSP, preliminary evidence suggests that the rates and subtypes of PSP may differ.^68^ Future research may benefit from evaluating whether the effect of tAN varies between hemorrhagic and ischemic stroke populations. Second, this study was limited to the short-term effects of a single 30-minute tAN session with no additional follow-up. Evidence suggests increased tAN dose, either with higher frequency of stimulation or longer duration, are associated with greater improvements in motor function and durability of response when paired with rehabilitation.^69,70^ There is currently limited research guiding optimal dosing parameters for tAN in PSP.^24,67^ As such, future clinical studies should explore optimal dosing and the durability of treatment effects to support the development of tAN as a therapeutic or rehabilitative intervention for PSP. Lastly, this study focused solely on behavioral outcome measures. Incorporating neuroimaging techniques in future research would allow the investigation of brain circuit changes associated with tAN, thereby providing a deeper understanding of its underlying analgesic mechanisms.

## CONCLUSIONS

In conclusion, although preliminary, our findings from this pilot study suggest that tAN is safe and feasible in patients with chronic PSP, and even a single session of tAN can improve pain threshold, making it a promising, noninvasive intervention for PSP management. Its ease of use, safety, and compatibility with at-home administration make it particularly well-suited for individuals with motor impairments or limited access to in-person care. Furthermore, unlike pharmacologic treatments, tAN poses no risk of polypharmacy or misuse and offers the potential for personalized stimulation protocols. Future larger-scale trials are needed to validate these findings and optimize treatment strategies for chronic PSP.

## Supplementary Material

1

To access the supplementary material accompanying this article, visit the online version of *Neuromodulation: Technology at the Neural Interface* at www.neuromodulationjournal.org and at https://doi.org/10.1016/j.neurom.2025.12.005.

## Figures and Tables

**Table 1.** Demographics and Clinical Characteristics of Patients With Chronic PSP.

Characteristics	Active group (*n* = 7)	Sham group (*n* = 8)	*p*-Value
Age (y)	51.43 ± 9.88	61.38 ± 8.43	0.84
Sex			0.28
Male	1 (14.3)	4 (50)	
Female	6 (85.7)	4 (50)	
Race			0.27
White	1 (14.3)	4 (50)	
African American	5 (71.4)	3 (37.5)	
Hispanic	0 (0)	1 (12.5)	
Asian	1 (14.3)	0 (0)	
Education (y)	15 ± 3.27	14.63 ± 2.62	0.81
Stroke type			1
Ischemic	6 (85.7)	6 (75)	
Hemorrhagic	1 (14.3)	2 (25)	
Stroke hemisphere			1
Right	0 (0)	0 (0)	
Left	7 (100)	8 (100)	
Stroke onset (y)	7.0 ± 5.23	5.25 ± 3.77	0.47
Pain onset after stroke (mo)	1.14 ± 1.46	0.63 ± 0.92	0.43
PEG score	5.71 ± 2.15	4.96 ± 2.26	0.52
Perception threshold (mA)			0.20
Cymba	1.66 ± 0.77	N/A	
Trigeminal	1.84 ± 0.84	N/A	
Earlobe	N/A	1.16 ± 0.45	

Data are expressed as mean ± SD or *n* (%). To assess between-group differences, two-sample *t*-tests were conducted for age, years of education, time since stroke onset, time of pain onset after stroke, and EPG scores. χ^2^ or Fisher exact tests were used to compare categorical variables, including sex, race, stroke type, and stroke hemisphere. A one-way ANOVA was used to compare PTs between groups.

PEG, Pain, Enjoyment of Life, and General Activity.
