# Supplementary material for: Transcutaneous Auricular Neurostimulation Modulates Pain Perception in Survivors of Stroke With Chronic Upper-Extremity Pain: A Randomized, Sham-Controlled Pilot Study
Source: Neuromodulation. Author manuscript; Available in PMC 2026 Jul 15. (PMC13371169; doi:10.1016/j.neurom.2025.12.005)
Supplement: 1 [file NIHMS2190314-supplement-1.pdf]

Supplementary Materials for

Transcutaneous Auricular Neurostimulation (tAN) Modulates Pain Perception in Stroke Survivors with Chronic Upper-Extremity Pain: A Randomized, Sham-Controlled Pilot Study

Xiaolong Peng, Stewart S. Cox, Brenna Baker-Vogel, Fisayo Omonije, Katherine Tucker, Bailey Huttig, Falon Sutton, Nicole Cash, Marion Wood, Steven A. Kautz, Bashar W. Badran, Jeffrey J. Borckardt

<u>This PDF file includes three+ supplemental figures referenced in the manuscript:</u>

Figure S1
Figure S2
Figure S3

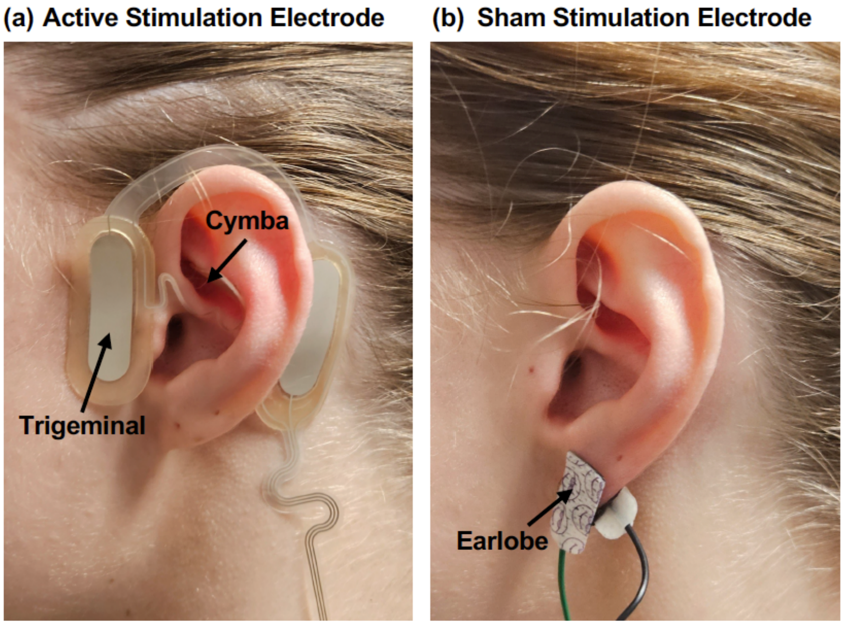

Figure S1. Electrode placement. (A) Active stimulation used hydrogel/hydrocolloid plastic electrodes (Spark Biomedical, Dallas, TX) to stimulate both the cymba conchae and the trigeminal sites. (B) Sham stimulation used silver/silver-chloride plastic electrodes (Neotech Products, Valencia, CA) to stimulate the earlobe site.

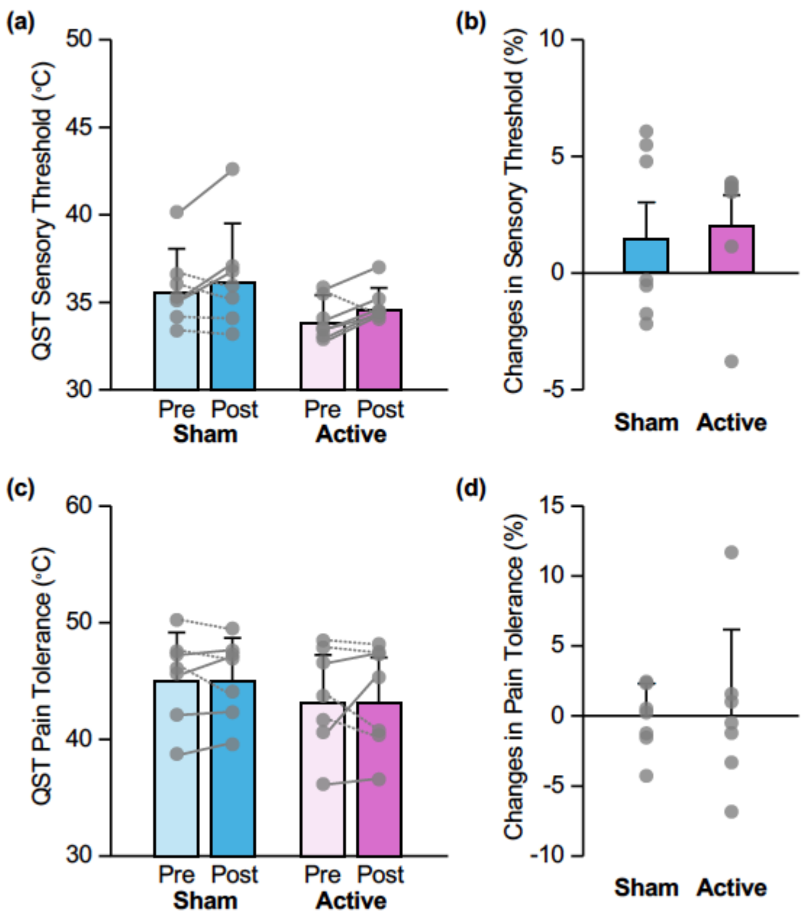

Figure S2. No significant change in sensory threshold or pain tolerance following tAN. (A) Repeated-measures two-way ANOVA of thermal sensory threshold revealed a significant main effect of time [$F(1,12) = 4.782$, $p = 0.042$], but no significant effect of group [$F(1,12) = 2.713$, $p = 0.126$] or group x time interaction [$F(1,12) = 0.042$, $p = 0.841$]. (B) There was no significant difference between the active and sham groups in the percent change in sensory threshold following tAN stimulation [$t(12) = 0.346$, $p = 0.734$]. (C) Additionally, analysis of thermal pain tolerance showed no significant main effects of group [$F(1,12) = 0.682$, $p = 0.425$], time [$F(1,12) < 0.001$, $p = 0.989$], or group x time interaction [$F(1,12) = 0.048$, $p = 0.831$], and (D) no difference was seen in the percent change between groups following stimulation [$t(12) = 0.234$, $p = 0.819$]. Solid and dashed lines indicate increased or decreased QST pain thresholds, respectively.

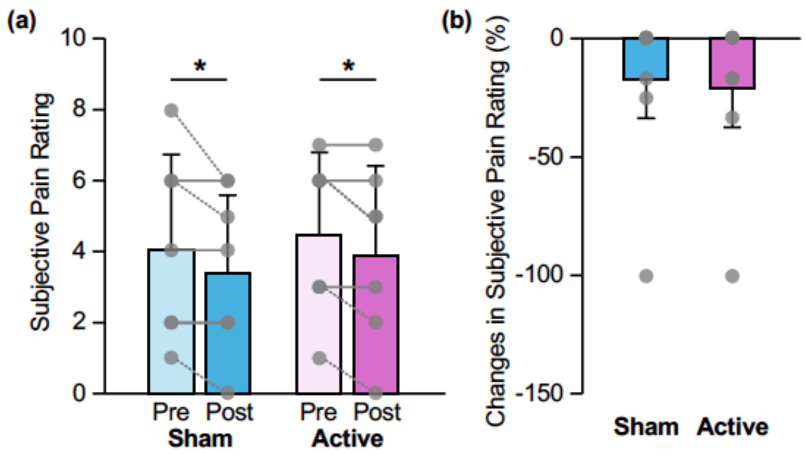

Figure S3. Subjective pain rating decreased after tAN in both the active and sham groups. (A) A repeated-measures two-way ANOVA of subjective pain ratings pre- and post-tAN revealed a significant main effect of time [F (1,12) = 10.110, p = 0.008], but no significant effect of group [F (1,12) = 0.114, p = 0.742] or "group x time" interaction [F (1,12) < 0.001, p > 0.999]. (B) A two-sample t-test comparing the percent change in subjective pain ratings from pre- to post-tAN showed no significant difference between the active and sham groups [t (12) = 0.185, p = 0.857]. Solid and dashed lines indicate unchanged or decreased QST pain thresholds, respectively. * p < 0.05.
